# Supplementary material for: Emergence of enhancers at late DNA replicating regions
Source: Nat Commun. 2024 Apr 24;15:3451. doi: 10.1038/s41467-024-47391-5 (PMC11043393; doi:10.1038/s41467-024-47391-5)
Supplement: Supplementary file 1 — Supplementary Information [file 41467_2024_47391_MOESM1_ESM.pdf]

Supplemental Material

Index

Supplemental Methods....3

Supplementary Figure 1. Log odds ratio of the conditional probability of recent liver enhancers dependent on replication time....4

Supplementary Figure 2. Scatterplot of mouse enhancers turnover and somatic replication time 5

Supplementary Figure 3. Relationship between mouse enhancer turnover and somatic replication time 6

Supplementary Figure 4. Mean DNA replication time versus enhancer turnover rate for mouse recent enhancers that align and do not align to the human genome. 7

Supplementary Figure 5. Correlation between mean mouse germ line replication time and TE turnover rate....8

Supplementary Figure 6. Enhancer turnover rate versus mean DNA replication time for mouse enhancers that overlap and do not overlap TE 9

Supplementary Figure 7. Singleton recent enhancers are less likely to overlap repetitive elements 10

Supplementary Figure 8. Increasing the number of species used in the definition of evolutionary conserved peaks produced similar outcomes 11

Supplementary Figure 9. TF binding prediction of human enhancers and orthologous non-enhancer sequences in mouse....12

Supplementary Figure 10. TF binding prediction of mouse enhancers and orthologous non-enhancer sequences in human....13

Supplementary Figure 11. Frequency of rare and common variants at human liver promoters and enhancers....14

Supplementary Figure 12. Distribution of fruit fly developmental and housekeeping enhancers....15

Supplementary Figure 13. Transcription factor binding sites enriched in early versus late replicating human liver enhancers 16

Supplementary Figure 14. Relative enrichment of homeo domain factors at early and late replicating human liver enhancers. 18

Supplementary Figure 15. Nucleotide composition analyses at mouse enhancers based on DNA replication time 19

Supplementary Figure 16. Nucleotide composition analyses at human liver promoters based on DNA replication time 20

Supplementary Figure 17. Gaussian mixture models of in vivo TF binding data in the cell line K562 21

Supplementary Figure 18. Enhancer turnover in cancer is associated with DNA replication time 23

Supplementary Figure 19. Replication time in prostate enhancers matched to recombination breakpoints 24

Supplementary Figure 20. Enhancer mutations in individual cancer samples 25

Supplementary Figure 21. Model performance on validation datasets 26

Supplementary Figure 22. Held-out Test performance of the trained models 27

Supplementary Tables 28

Supplementary Table 1. Fisher test for the differential overlap of recent and conserved enhancers with accessible genomic regions 28

Supplementary Table 2. Number of enhancers and mutations per cancer type 29

Supplementary Table 3. Kruskal-Wallis' tests output for the difference in GC% across DNA replication time quintiles in Fig. 5A 30

Supplementary Table 4. Number of regions per DNA replication time quintile in Fig. 5B 30

Supplementary Table 5. Number of enhancers in Fig. 6 30

Supplementary Table 6. Mean human and mouse enhancers width 32

References 33

Supplemental Methods

Data processing for constructing Figure 2B

We highlighted specific sequence segments to visualize the motif positions identified by FIMO within the candidate enhancer sequences. For this visualization, we leveraged the R package ggmsa (version 1.3.4) (Zhou et al. 2022). Of note, the segments of putative non-enhancer sequences illustrated in the figure did not yield any instances of mouse or human CEBPA or HNF4A motifs.

The complete sequence of mouse and human candidate enhancers was mapped to either the human or mouse genomes using the UCSC liftOver tool with a minimum ratio of bases that remap (-minMatch) of 0.6. We utilized the mm10 genome assembly for mouse and hg38 for human. To refine the dataset, we identified the mouse and human candidate enhancers overlapping CEBPA or HNF4A binding sites as identified by ChIP-Seq data specific to each species (Schmidt et al. 2010).

We focused on the candidate enhancers overlapping with either CEBPA or HNF4A binding sites for subsequent analysis. We used the FIMO tool to identify CEBPA and HNF4A motif instances in the candidate enhancers overlapping CEBPA or HNF4A binding sites, respectively (Grant et al. 2011). In this analysis, we employed mouse CisBP-2.0 motifs for the mouse candidate enhancers and human CisBP-2.0 motifs for the human candidate enhancers (Weirauch et al. 2014). FIMO was used with default parameters, using a p-value threshold of $<= 1 \times 10^{-04}$.

To prioritize candidate enhancers, we ranked them based on their CisBP-2.0 motif scores. Specifically, we selected the candidate enhancer with the highest motif score for each transcription factor and species, provided it surpassed the prediction threshold in the domain adaptive model (predicted probability ≥ 0.9).

The full sequences of the chosen mouse and human candidate enhancers, along with their corresponding mappings to the human or mouse genome, were aligned using Clustal Omega (version 1.2.4) via the EMBL-EBI tool with default parameters (Madeira et al. 2022). The clustalW output format was converted to FASTA format using the EMBOSS Seqret tool (Madeira et al. 2022).

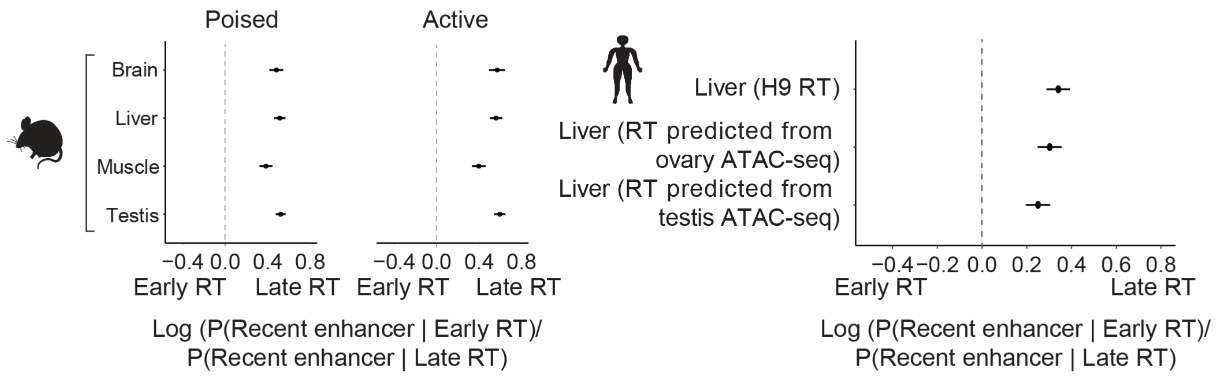

Supplementary Figure 1. Log odds ratio of the conditional probability of recent liver enhancers dependent on replication time

P(Recent enhancer | Early RT) / P(Recent enhancer | Late RT) values for mouse tissue-specific enhancers separated into poised and active (left panel). Dots represent the mean conditional probability, and error bars are standard errors. Human germline DNA replication time was predicted from ovary and testis ATAC-seq. Icons from BioRender.com.

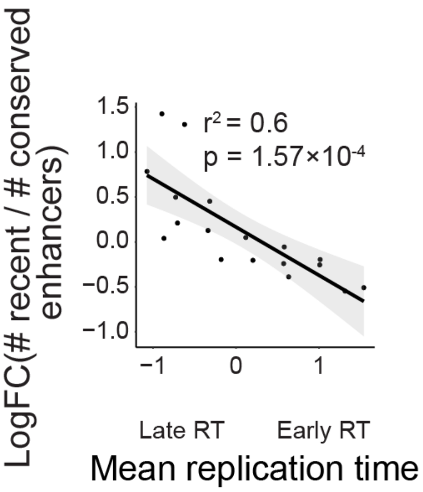

Supplementary Figure 2. Scatterplot of mouse enhancers turnover and somatic replication time

Scatterplot of mean somatic replication time across the 18 clusters shown in **(Fig. 1C)**. R^2 and p-value are indicated. Shaded area represents 95% confidence interval of the best fit. Mean somatic replication time is calculated across 22 cell lines (**Methods**).

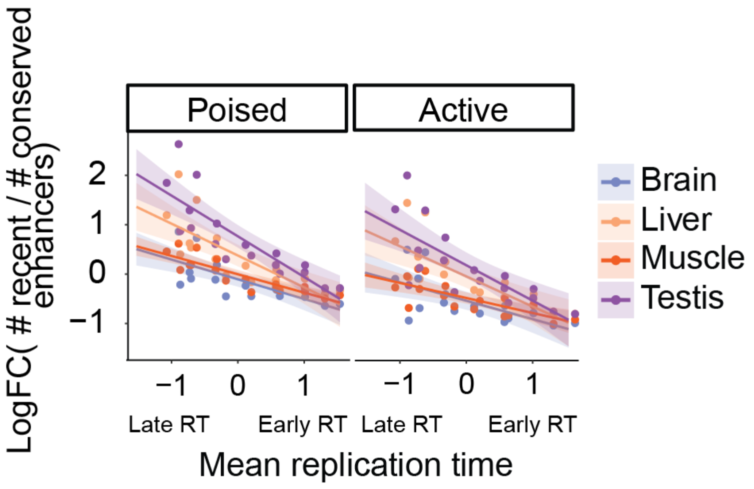

Supplementary Figure 3. Relationship between mouse enhancer turnover and somatic replication time

Scatterplot showing somatic cell lines mean replication time (x-axis) and enhancer turnover (log FC of recent enhancers against conserved enhancers) by tissue and enhancer type (poised or active) (lm for the difference in slopes between liver and testis against brain and muscle, formula = logFC.enh ~ mean_rt + tissue_pair + tissue.pair:mean_rt , where logFC.enh is the value of log (number of recent / number of conserved enhancers), tissue_pair is either “liver_testis” or “brain_muscle” codified as binary, 1 and 0, respectively, and mean_rt is the mean of developmental cell types’ replication time, *t* = 2.26 and *t* = 1.86 for poised and active enhancers, respectively, p = 3.1×10^{-02} and p = 7.25×10^{-02}, for poised and active enhancers). Shaded areas represent 95% confidence interval of the best fit.

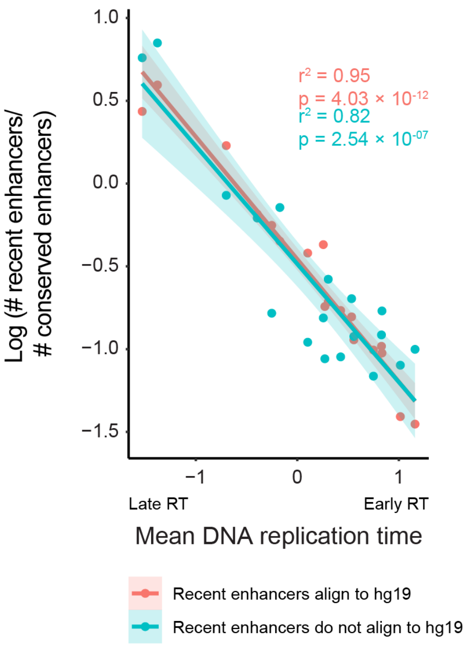

Supplementary Figure 4. Mean DNA replication time versus enhancer turnover rate for mouse recent enhancers that align and do not align to the human genome. The enhancer turnover rate was calculated using mouse recent enhancers that align and do not align to the human genome (hg19) (liftOver -minMatch = 0.6) for each cluster shown in **Fig. 1C**. Mean replication time represents mean germ line DNA replication time. R^2 and p-value are displayed for each group. Shaded areas represent the 95% confidence interval of the best fits.

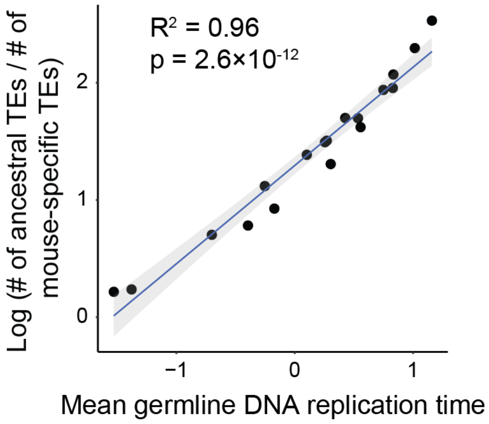

Supplementary Figure 5. Correlation between mean mouse germ line replication time and TE turnover rate

TE turnover rate and mean germ line DNA replication time are shown for the 18 clusters shown in **Fig. 1C**. TE turnover rate was defined as log (number of ancestral TEs / number of mouse-specific TEs). R^2 and p-value are shown. Shaded area represents 95% confidence interval of the best fit.

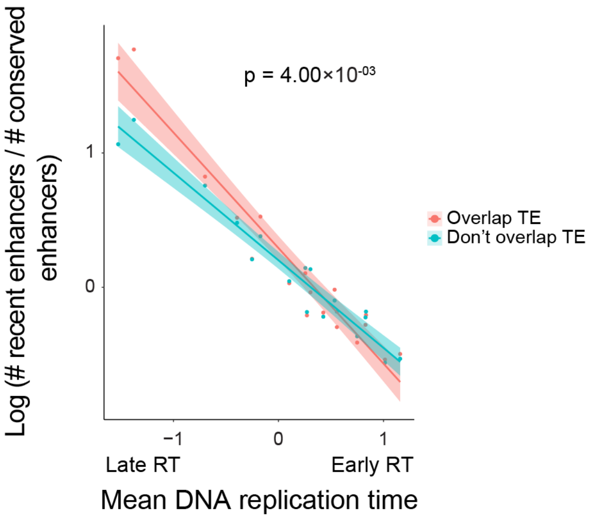

Supplementary Figure 6. Enhancer turnover rate versus mean DNA replication time for mouse enhancers that overlap and do not overlap TE

Mean germ line DNA replication time against mean enhancer turnover rate (defined as log (number of recent enhancers / number of conserved enhancers)) across 18 DNA replication time clusters as defined in **Fig. 1C**. Mean enhancer turnover rates and DNA replication time values were separated for enhancers overlapping TE and not overlapping TE (shown in red and blue, respectively). The difference in slope between the two groups was not significant (ANCOVA, p-value shown in the figure). R^2 = 0.94 (p-value = 4.12×10^{-11}) and 0.95 (p-value = 1.37×10^{-11}) for enhancers overlapping and not overlapping TE, respectively. Shaded areas represent the 95% confidence interval of the best fits.

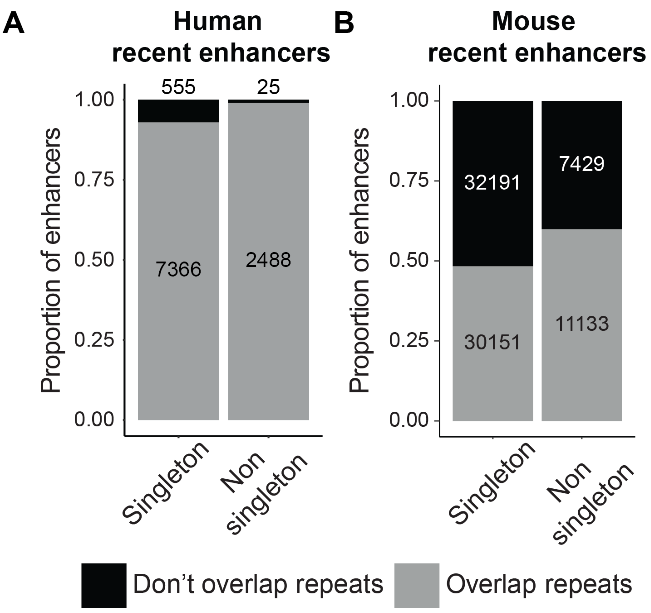

Supplementary Figure 7. Singleton recent enhancers are less likely to overlap repetitive elements

(A) Proportion of human recently evolved enhancers overlapping repetitive elements (Fisher's exact test, $p = 3.56 \times 10^{-40}$, odds ratio = 0.13). Enhancers are divided into singleton and non-singleton based on cluster analysis (**Methods**). **(B)** Same as in **(A)** for mouse recent enhancers (Fisher's exact test, $p = 6.8 \times 10^{-171}$, odds ratio = 0.63).

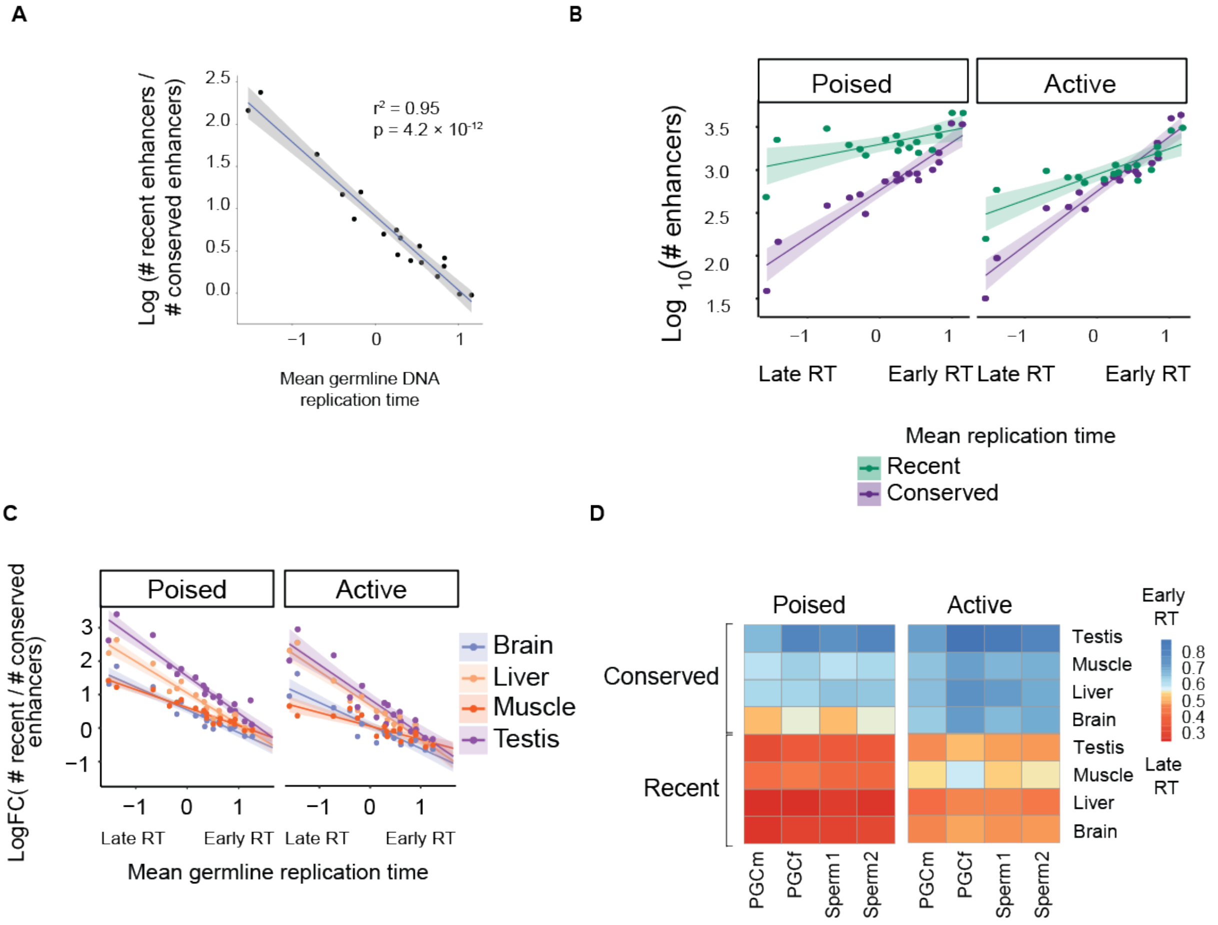

Supplementary Figure 8. Increasing the number of species used in the definition of evolutionary conserved peaks produced similar outcomes

Evolutionarily conserved enhancers are defined by cross-species conservation among five species (including mouse) (n = 48580)**. (A)** Scatterplot of mean germline replication time (PGC + SSP) across the 18 clusters shown in **Fig. 1C**. R^2 and p-value are indicated. The shaded area represents the 95% confidence interval of the best fit. **(B)** Scatterplot of germline mean DNA replication time (PGC + SSP) and $\log_{10}$-transformed numbers of recent and conserved enhancers. Each data point represents a cluster as defined in **Fig. 1C**. The shaded areas represent the 95% confidence interval of the best fits. **(C)** Scatterplots of germline mean DNA replication time (PGC + SSP) and enhancer turnover by tissue and enhancer type. Each data instance corresponds to a cluster in **Fig. 1C**. The shaded areas represent the 95% confidence interval of the best fits. (**D)** Heatmaps of mean PGC and SSC DNA replication time of poised and active mouse enhancers separated by tissue and type.

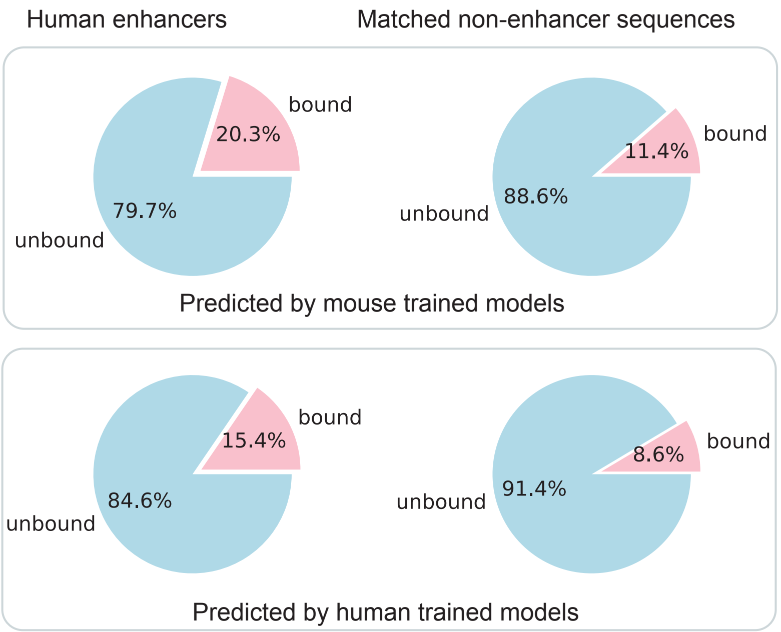

Supplementary Figure 9. TF binding prediction of human enhancers and orthologous non-enhancer sequences in mouse

The pink sections represent the proportions of human enhancers or non-functional regions predicted to be bound by CEBPA or HNF4A, while the blue sections represent regions not predicted to be bound by any of the TFs. Predictions from models trained on mouse data are displayed on the top, while the bottom row shows predictions from models trained on human data.

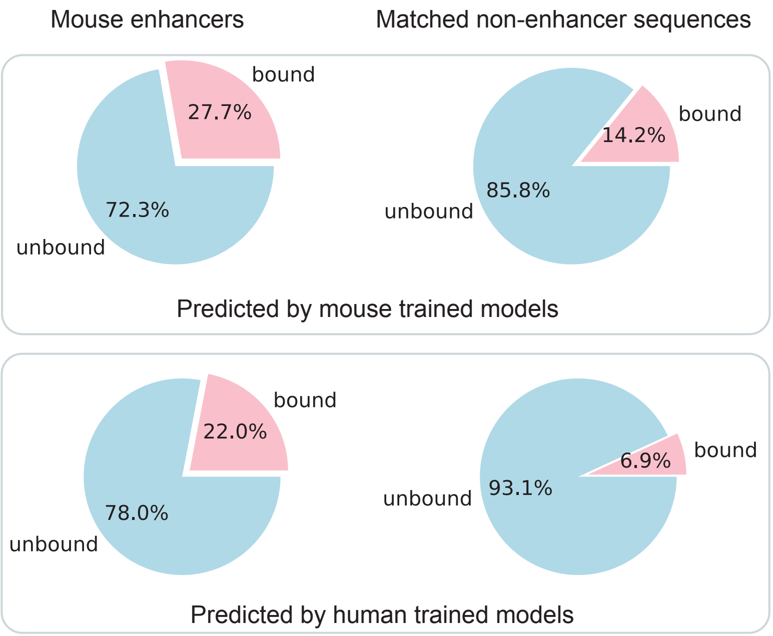

Supplementary Figure 10. TF binding prediction of mouse enhancers and orthologous non-enhancer sequences in human

The pink sections represent the proportions of mouse enhancers or non-functional regions predicted to be bound by CEBPA or HNF4A, while the blue sections represent regions not predicted to be bound by any of the TFs. Predictions from models trained on mouse data are displayed on the top, while the bottom row shows predictions from models trained on human data.

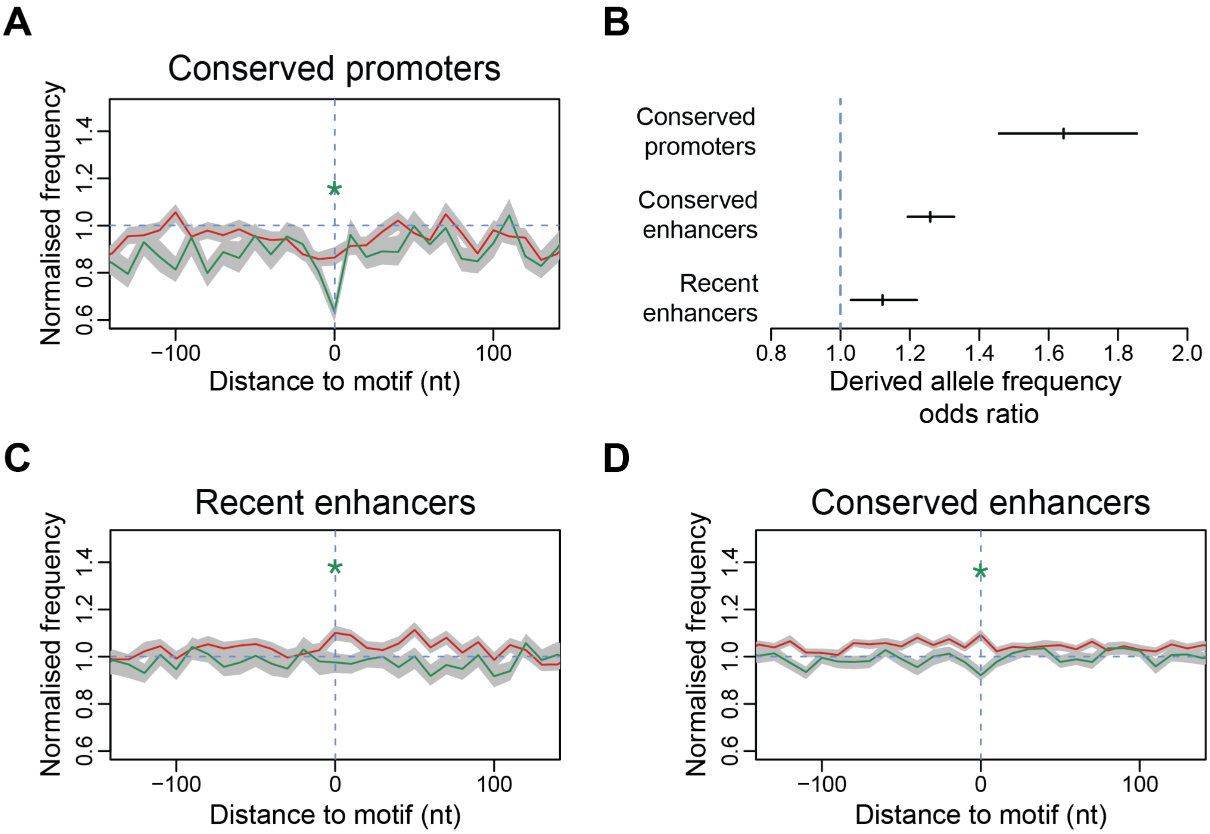

Supplementary Figure 11. Frequency of rare and common variants at human liver promoters and enhancers

(A) Frequency of rare (<1.5% population frequency, red) and common (>5% frequency, green) SNPs at conserved human liver promoters. Promoters were centred based on the positions of functional liver motifs (10bp windows). Allele frequencies were normalised by the average frequencies within 2-4 kb upstream and downstream flanking regions for each category. Shaded areas represent 95% confidence interval obtained by sampling the data with replacement ("*" indicates the absence of overlap of rare and common alleles' confidence intervals). **(B)** Odds Ratio of Derived allele frequency (DAF) of liver promoters and enhancers. Vertical bars represent DAF odds ratio at recent and conserved enhancers and conserved promoters centred on functional liver motifs (10bp windows) compared to a similar number of windows selected at random from the genome (relative to **Fig.3 A-C**). Error bars represent 95% confidence interval obtained from a Fisher's exact test. **(C-D)** Similar to **(A)**, the frequency of rare (red) and common (green) allele frequencies at recent **(C)** and conserved **(D)** liver enhancers.

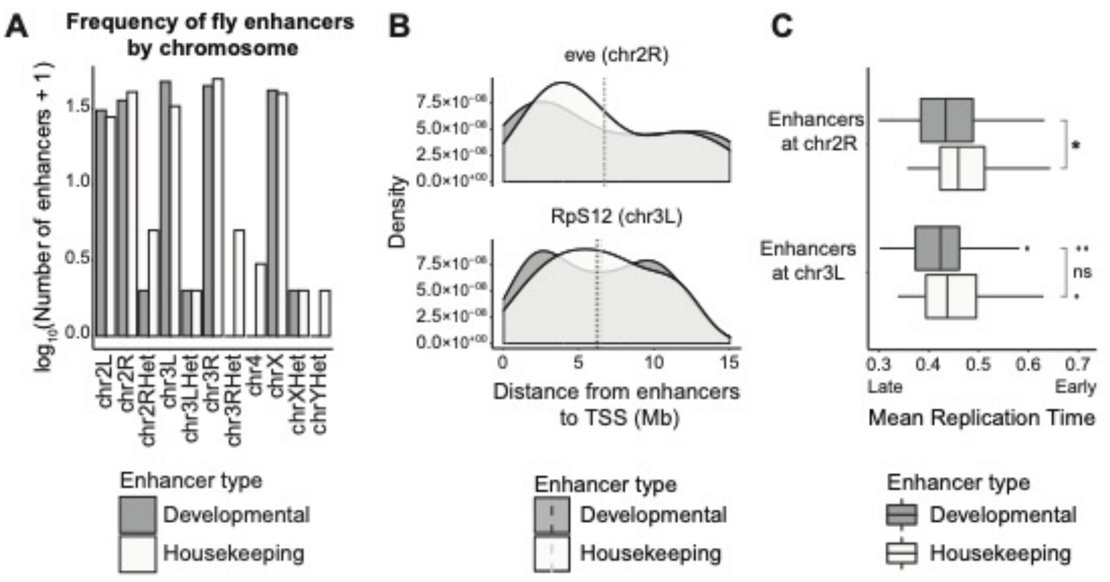

Supplementary Figure 12. Distribution of fruit fly developmental and housekeeping enhancers

(A) Frequency of developmental and housekeeping fly enhancers across the *Drosophila* genome (dm3). **(B)** Distance of developmental and housekeeping enhancers to the Transcription Start Sites (TSSs) of *eve* and *RpS12*, whose promoters were used as developmental and housekeeping promoters, respectively (n = 35 and 40 developmental and housekeeping enhancers at chr2R, n = 47 and 32 developmental and housekeeping enhancers at chr3L). **(C)** Mean replication time of enhancers shown in **(B)** **(**Mann-Whitney *U*-test, developmental vs housekeeping enhancers, significance code 'ns' P > 0.05 and '*' P ≤ 0.05**)**.

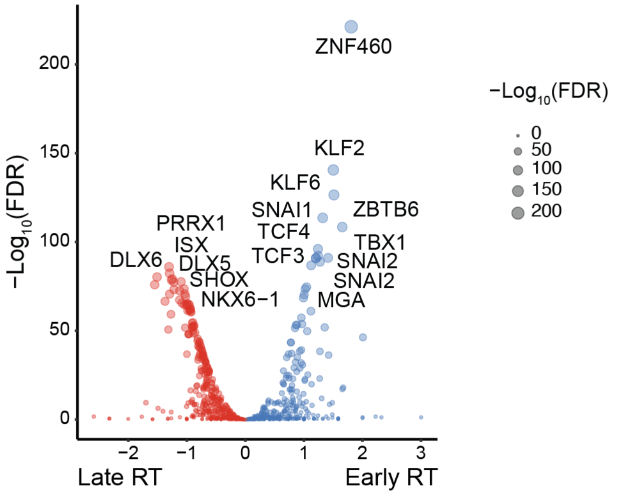

Supplementary Figure 13. Transcription factor binding sites enriched in early versus late replicating human liver enhancers

Enriched JASPAR motifs between early and late replicating liver enhancers. X-axis shows the relative enrichment for each motif at early versus late replicating enhancers; y-axis represent -$\log_{10}$ (Fisher's exact test, FDR).

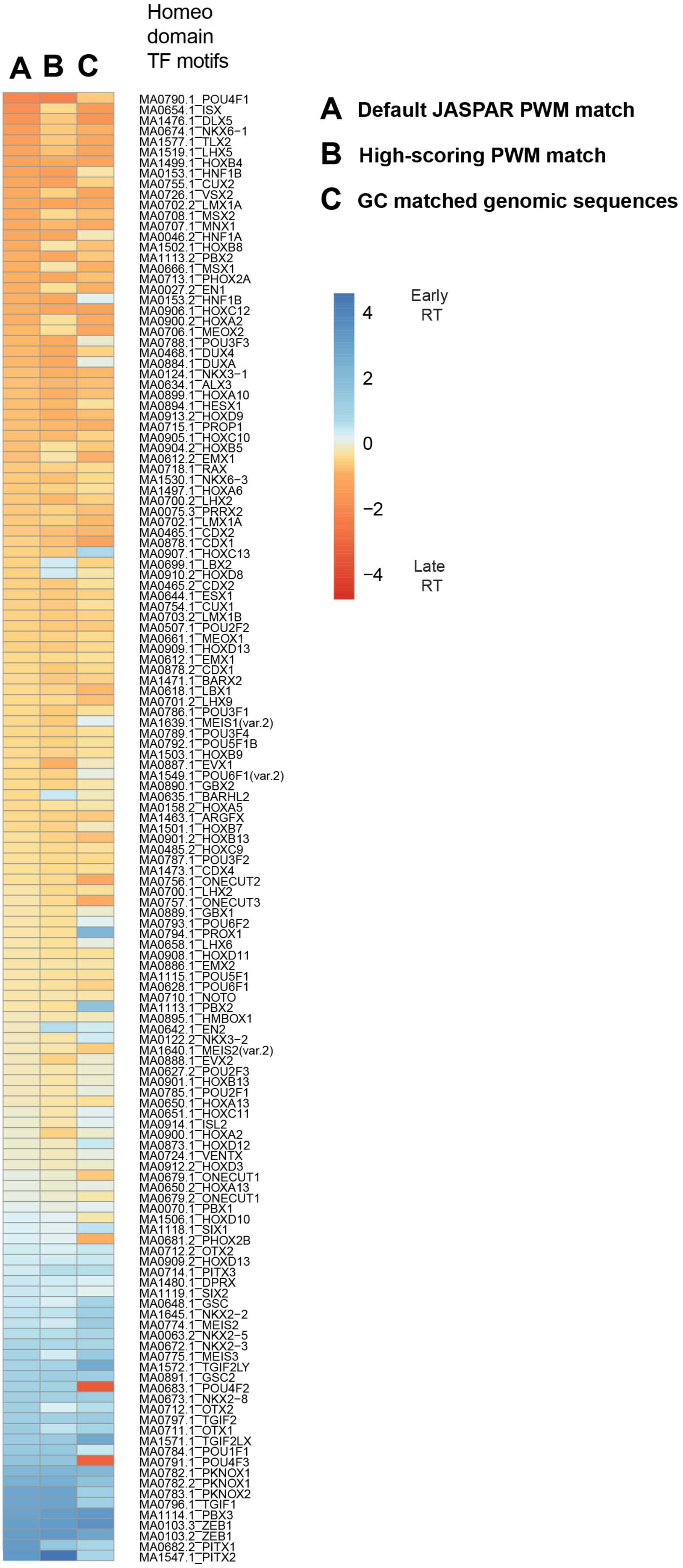
Homeo domain TF motifs
A B C
A Default JASPAR PWM match
B High-scoring PWM match
C GC matched genomic sequences
Early RT
4
2
0
−2
−4
Late RT
MA0790.1_POU4F1
MA0654.1_ISX
MA1476.1_DLX5
MA0674.1_NKX6−1
MA1577.1_TLX2
MA1519.1_LHX5
MA1499.1_HOXB4
MA0153.1_HNF1B
MA0755.1_CUX2
MA0726.1_VSX2
MA0702.2_LMX1A
MA0708.1_MSX2
MA0707.1_MNX1
MA0046.2_HNF1A
MA1502.1_HOXB8
MA1113.2_PBX2
MA0666.1_MSX1
MA0713.1_PHOX2A
MA0027.2_EN1
MA0153.2_HNF1B
MA0906.1_HOXC12
MA0900.2_HOXA2
MA0706.1_MEOX2
MA0788.1_POU3F3
MA0468.1_DUX4
MA0884.1_DUXA
MA0124.1_NKX3−1
MA0634.1_ALX3
MA0899.1_HOXA10
MA0894.1_HESX1
MA0913.2_HOXD9
MA0715.1_PROP1
MA0905.1_HOXC10
MA0904.2_HOXB5
MA0612.2_EMX1
MA0718.1_RAX
MA1530.1_NKX6−3
MA1497.1_HOXA6
MA0700.2_LHX2
MA0075.3_PRRX2
MA0702.1_LMX1A
MA0465.1_CDX2
MA0878.1_CDX1
MA0907.1_HOXC13
MA0699.1_LBX2
MA0910.2_HOXD8
MA0465.2_CDX2
MA0644.1_ESX1
MA0754.1_CUX1
MA0703.2_LMX1B
MA0507.1_POU2F2
MA0661.1_MEOX1
MA0909.1_HOXD13
MA0612.1_EMX1
MA0878.2_CDX1
MA1471.1_BARX2
MA0618.1_LBX1
MA0701.2_LHX9
MA0786.1_POU3F1
MA1639.1_MEIS1(var.2)
MA0789.1_POU3F4
MA0792.1_POU5F1B
MA1503.1_HOXB9
MA0887.1_EVX1
MA1549.1_POU6F1(var.2)
MA0890.1_GBX2
MA0635.1_BARHL2
MA0158.2_HOXA5
MA1463.1_ARGFX
MA1501.1_HOXB7
MA0901.2_HOXB13
MA0485.2_HOXC9
MA0787.1_POU3F2
MA1473.1_CDX4
MA0756.1_ONECUT2
MA0700.1_LHX2
MA0757.1_ONECUT3
MA0889.1_GBX1
MA0793.1_POU6F2
MA0794.1_PROX1
MA0658.1_LHX6
MA0908.1_HOXD11
MA0886.1_EMX2
MA1115.1_POU5F1
MA0628.1_POU6F1
MA0710.1_NOTO
MA1113.1_PBX2
MA0895.1_HMBOX1
MA0642.1_EN2
MA0122.2_NKX3−2
MA1640.1_MEIS2(var.2)
MA0888.1_EVX2
MA0627.2_POU2F3
MA0901.1_HOXB13
MA0785.1_POU2F1
MA0650.1_HOXA13
MA0651.1_HOXC11
MA0914.1_ISL2
MA0900.1_HOXA2
MA0873.1_HOXD12
MA0724.1_VENTX
MA0912.2_HOXD3
MA0679.1_ONECUT1
MA0650.2_HOXA13
MA0679.2_ONECUT1
MA0070.1_PBX1
MA1506.1_HOXD10
MA1118.1_SIX1
MA0681.2_PHOX2B
MA0712.2_OTX2
MA0909.2_HOXD13
MA0714.1_PITX3
MA1480.1_DPRX
MA1119.1_SIX2
MA0648.1_GSC
MA1645.1_NKX2−2
MA0774.1_MEIS2
MA0063.2_NKX2−5
MA0672.1_NKX2−3
MA0775.1_MEIS3
MA1572.1_TGIF2LY
MA0891.1_GSC2
MA0683.1_POU4F2
MA0673.1_NKX2−8
MA0712.1_OTX2
MA0797.1_TGIF2
MA0711.1_OTX1
MA1571.1_TGIF2LX
MA0784.1_POU1F1
MA0791.1_POU4F3
MA0782.1_PKNOX1
MA0782.2_PKNOX1
MA0783.1_PKNOX2
MA0796.1_TGIF1
MA1114.1_PBX3
MA0103.3_ZEB1
MA0103.2_ZEB1
MA0682.2_PITX1
MA1547.1_PITX2

Supplementary Figure 14. Relative enrichment of homeo domain factors at early and late replicating human liver enhancers.

Similar to **Fig. 5E**. The left column of the heatmap shows the relative enrichment of homeodomain motifs at early and late replicating human liver enhancers. In the centre column only high scoring motifs are considered. The column on the right shows the relative enrichment of homeodomain factors in random regions of the genome matched by GC content. PWM IDs and transcription factor names are indicated. N = 139 homeo domain PWMs.

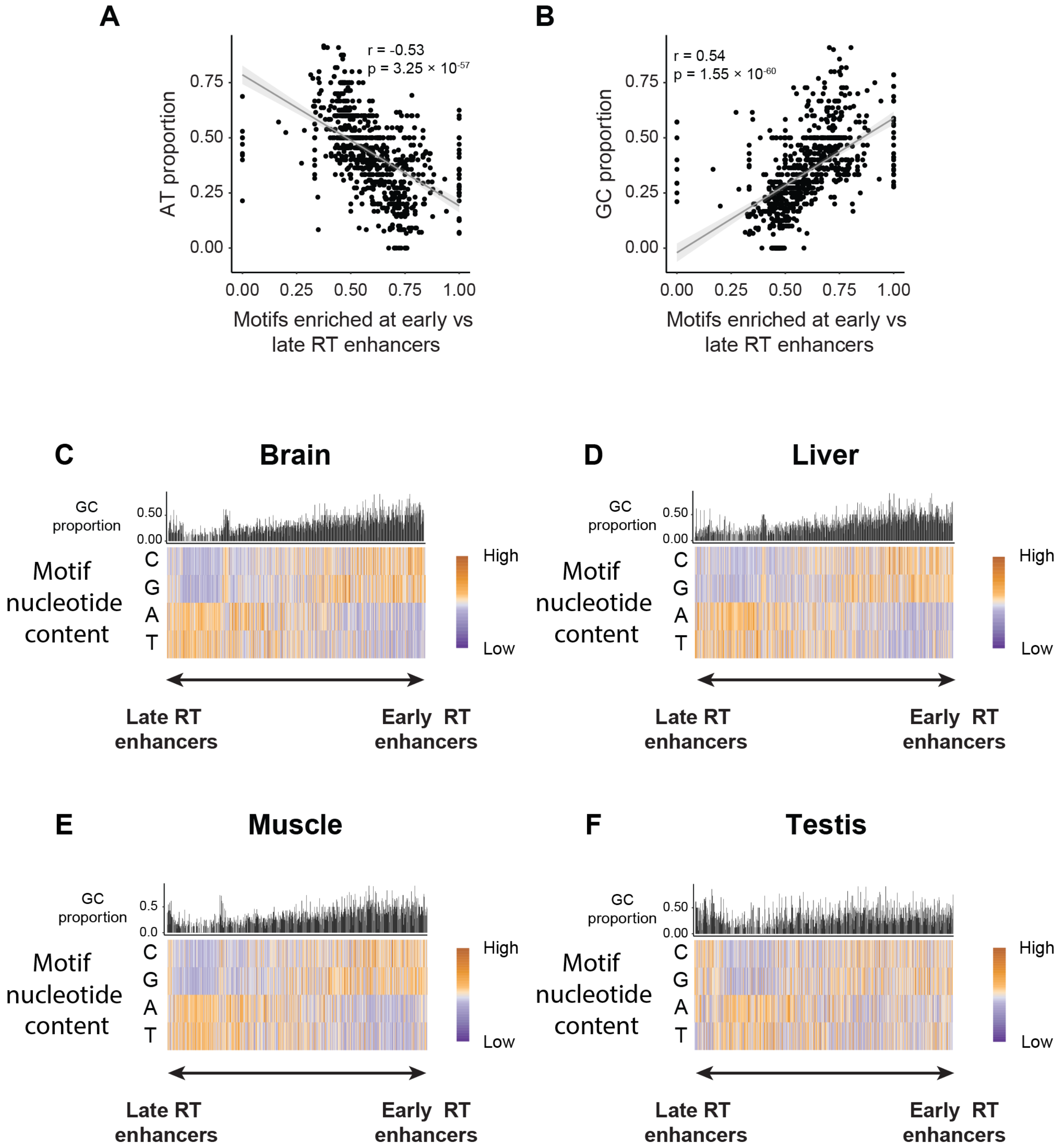

Supplementary Figure 15. Nucleotide composition analyses at mouse enhancers based on DNA replication time

(A-B) Correlation between AT **(A)** and GC **(B)** proportion of TF binding motifs enriched at early vs late replicating mouse enhancers. Pearson's r and p-value are indicated in each case. All tissues' enhancers were included (brain, liver, muscle and testis). The shaded areas represent the 95% confidence interval of the best fits. **(C-F)** Nucleotide composition of TF binding motifs enriched at early and late replicating mouse enhancers by tissue: **(C)** brain, **(D)** liver, **(E)** muscle and **(F)** testis (n = 6082, 6240, 2994 and 5094 brain, liver, muscle and testis enhancers, respectively; equal numbers of early RT and late RT enhancers for each tissue).

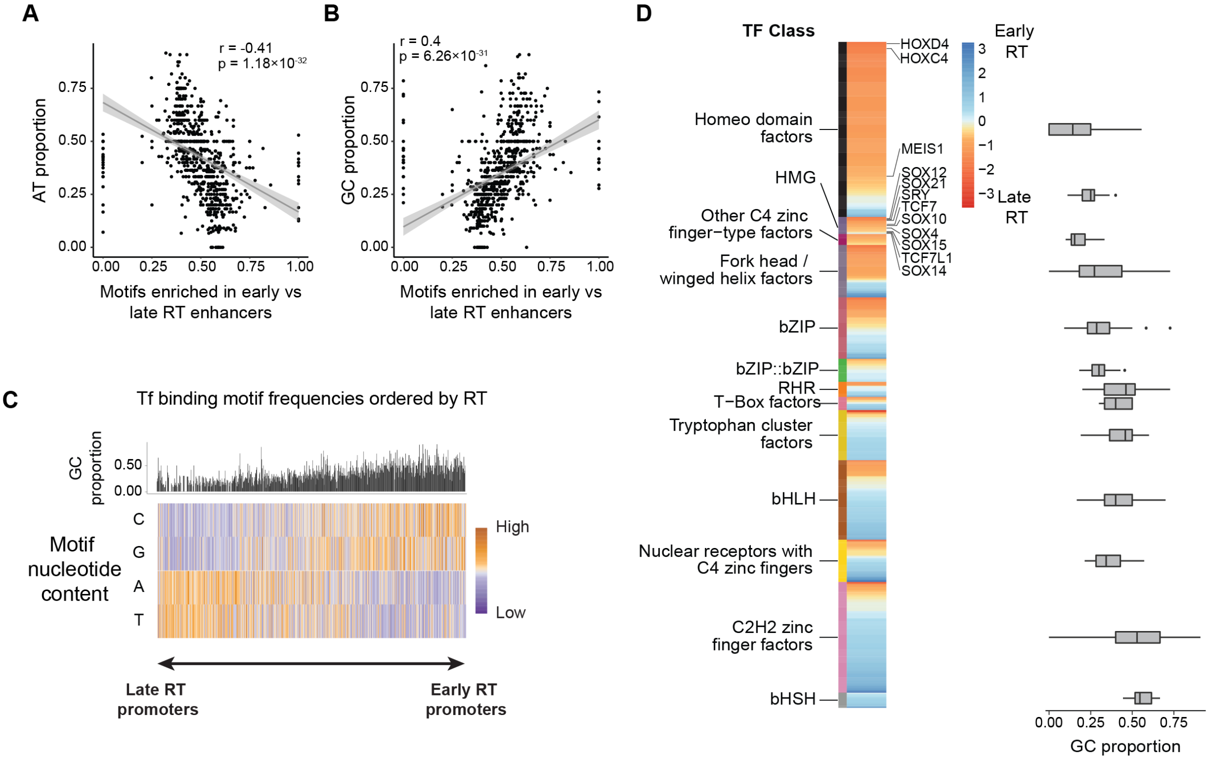

Supplementary Figure 16. Nucleotide composition analyses at human liver promoters based on DNA replication time

(A-B) Correlation between AT **(A)** and GC **(B)** proportion of TF binding sites and TF relative enrichment at early vs late replicating promoters. Pearson correlation r and p-value are shown in each case. The shaded areas represent the 95% confidence interval of the best fits. **(C)** Nucleotide proportion of TF binding motifs at early and late replicating human liver promoters (n = 2131 early RT enhancers and 2131 late RT enhancers). **(D)** Relative enrichment of TF binding motifs at early against late replicating promoters grouped by TF Class (left), some example motifs are indicated. The GC content of the motifs belonging to every TF Class is shown on the right (HMG = High-mobility group domain factors; bZIP = Basic leucine zipper factors; RHR = Rel homology region factors; bHLH = Basic helix-loop-helix factors; bHSH = Basic helix-span-helix factors). Only TF Classes with more than ten TFs are shown.

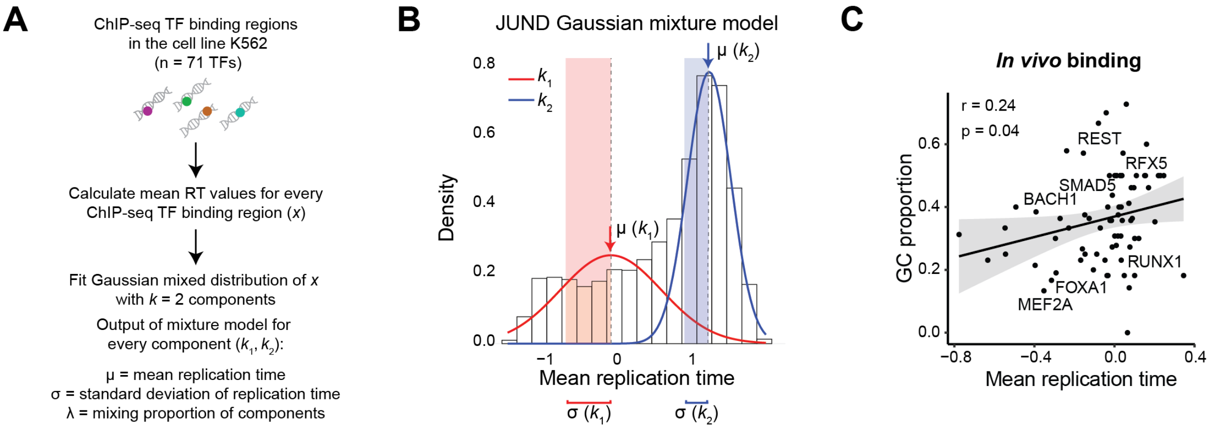

Supplementary Figure 17. Gaussian mixture models of in vivo TF binding data in the cell line K562

(A) Pipeline of Gaussian mixture models. A model with two components (k_1, k_2) was fitted for the binding sites' mean replication time of every TF (n = 71 TFs). After fitting a model, we got mean RT (μ), RT standard deviation (σ) and mixing proportion (λ) values for every component. **(B)** Example of mixture model (JUND). The distribution of JUND binding sites' mean replication time is shown with a histogram. The distribution of the components k_1 and k_2 according to the fitted mixture model are represented with a red and a blue line, respectively. The mean RT value of every distribution is noted with a colour matched arrow. The shaded areas represent one standard deviation from the mean RT values. **(C)** Scatterplot of DNA replication time versus GC content of ChIP-seq binding sites in the cell line K562 for 71 TF ChIP-seq datasets. Mean replication time of binding sites in the later replicating cluster for each TF is shown (**Methods**). Pearson correlation coefficient (r) and p-value are shown. Shaded region represents the 95% confidence interval of the line of best fit. Icon image from BioRender.com.

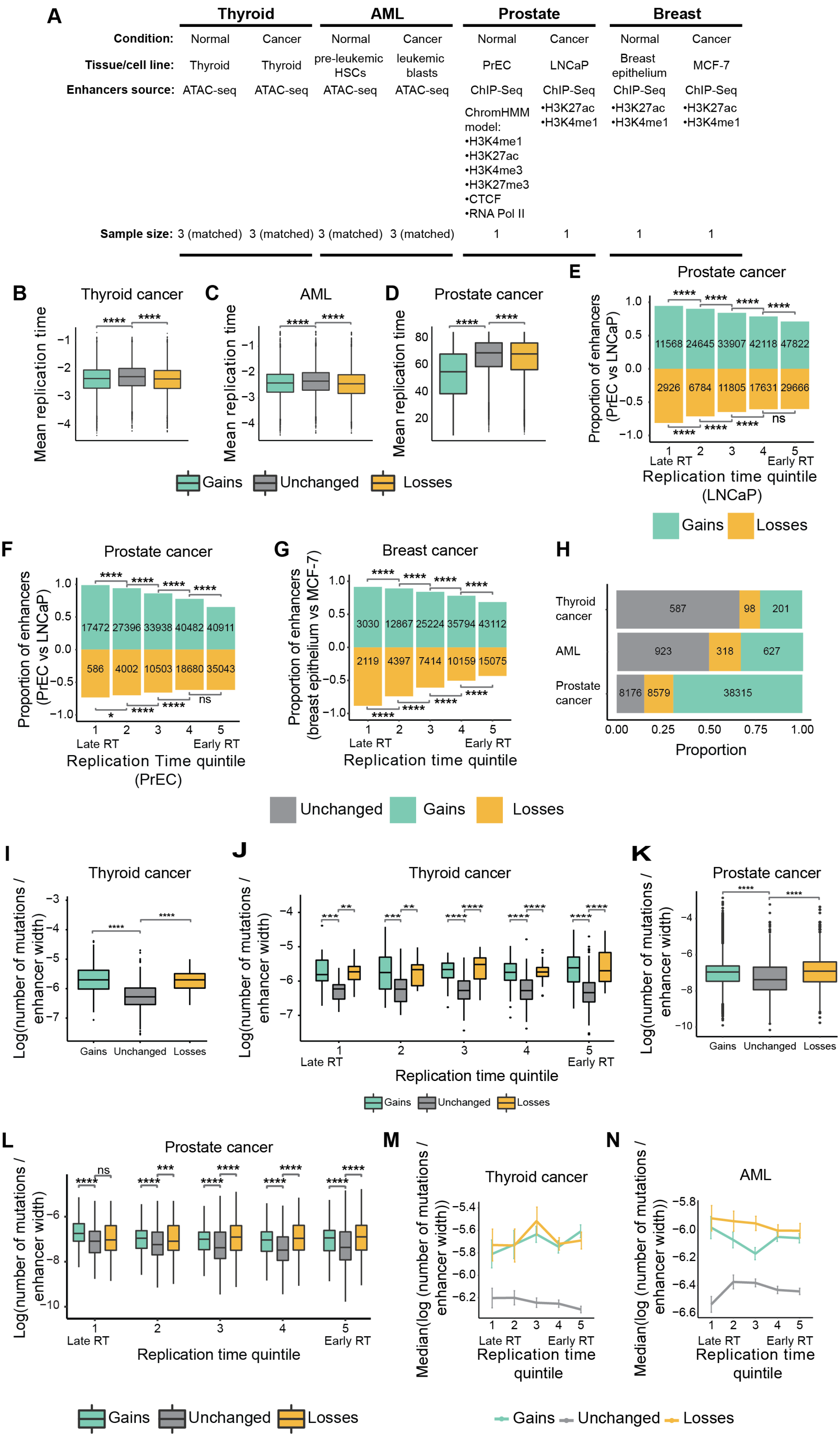
A
Thyroid
AML
Prostate
Breast
Condition: Normal Cancer Normal Cancer Normal Cancer Normal Cancer
Tissue/cell line: Thyroid Thyroid pre-leukemic HSCs leukemic blasts PrEC LNCaP Breast epithelium MCF-7
Enhancers source: ATAC-seq ATAC-seq ATAC-seq ATAC-seq ChIP-Seq ChIP-Seq ChIP-Seq ChIP-Seq
ChromHMM model: •H3K4me1 •H3K27ac •H3K4me3 •H3K27me3 •CTCF •RNA Pol II
•H3K27ac •H3K4me1
•H3K27ac •H3K4me1
•H3K27ac •H3K4me1
Sample size: 3 (matched) 3 (matched) 3 (matched) 3 (matched) 1 1 1 1
B
Thyroid cancer
Mean replication time
C
AML
Mean replication time
D
Prostate cancer
Mean replication time
E
Prostate cancer
Proportion of enhancers (PrEC vs LNCaP)
11568 24645 33907 42118 47822
2926 6784 11805 17631 29666
ns
Late RT
Early RT
Replication time quintile (LNCaP)
Gains Unchanged Losses
Gains Losses
F
Prostate cancer
Proportion of enhancers (PrEC vs LNCaP)
17472 27396 33938 40482 40911
586 4002 10503 18680 35043
ns
Late RT
Early RT
Replication Time quintile (PrEC)
G
Breast cancer
Proportion of enhancers (breast epithelium vs MCF-7)
3030 12867 25224 35794 43112
2119 4397 7414 10159 15075
Late RT
Early RT
Replication time quintile
H
Thyroid cancer
587 98 201
AML
923 318 627
Prostate cancer
8176 8579 38315
Proportion
Unchanged Gains Losses
I
Thyroid cancer
Log(number of mutations / enhancer width)
Gains Unchanged Losses
J
Thyroid cancer
Log(number of mutations / enhancer width)
Late RT
Early RT
Replication time quintile
K
Prostate cancer
Log(number of mutations / enhancer width)
Gains Unchanged Losses
Gains Unchanged Losses
L
Prostate cancer
Log(number of mutations / enhancer width)
ns
Late RT
Early RT
Replication time quintile
M
Thyroid cancer
Median(log (number of mutations / enhancer width))
Late RT
Early RT
Replication time quintile
N
AML
Median(log (number of mutations / enhancer width))
Late RT
Early RT
Replication time quintile
Gains Unchanged Losses
Gains Unchanged Losses

Supplementary Figure 18. Enhancer turnover in cancer is associated with DNA replication time

(A) Summary of the datasets used to define enhancers in normal cell types and tissues and their cancer counterparts. The number of samples is indicated. **(B-D)** Mean replication time values for gains, losses and unchanged enhancers in thyroid cancer **(B),** AML **(C)** and prostate cancer **(D).** n = 41046, 28426, and 34228 gains, unchanged enhancers, and losses for thyroid cancer. $p = 3.76 \times 10^{-70}$ and $p = 2.13 \times 10^{-86}$ for gains and losses versus unchanged enhancers in thyroid cancer. n = 58860, 45183, and 48244 gains, unchanged enhancers, and losses for AML. $p = 2.75 \times 10^{-109}$ and $p = 4.21 \times 10^{-205}$ for gains and losses versus unchanged enhancers for AML, respectively. n = 159204, 40658, and 68566 gains, unchanged enhancers, and losses for prostate cancer. $p < 2.2 \times 10^{-16}$ and $p = 7.51 \times 10^{-26}$ for the difference between gains and losses versus unchanged enhancers (two-sided Mann-Whitney *U*-test). **(E-G)** Proportion of gains and losses in prostate (based on LNCaP or PrEC replication time, **E** and **F**, respectively)**,** and breast cancer **(G)** across replication time quintiles (one-sided Fisher's exact test, alternative = "greater", compared to unchanged enhancers). $p = 7.69 \times 10^{-52}$, 1.79×10^{-114}, 7.2×10^{-100}, and 7.75×10^{-204} for the proportion of gains in consecutive quintiles in prostate cancer (panel E). $p = 2.46 \times 10^{-32}$, 2.78×10^{-29}, 8.59×10^{-20}, and 0.16 for the proportion of losses in consecutive quintiles in prostate cancer (panel E). p = 7.63 × 10-161, 1.06 × 10-263, 6.28 × 10-233, and $p < 2.2 \times 10^{-16}$ for the proportion of gains in consecutive quintiles (panel F). p = 0.03, 4.39 × 10-09, 6.38 × 10-18, and 0.47 for the proportion of losses in consecutive quintiles (panel F). $p = 4.66 \times 10^{-05}$, 2.23×10^{-50}, 2.07×10^{-93}, and 9.92×10^{-274} for the proportion of gains in consecutive quintiles in quintiles 1 to 5 (breast cancer). $p = 4.46 \times 10^{-51}$, 1.10×10^{-69}, 4.74×10^{-76}, and 6.14×10^{-59} for the proportion of losses in consecutive quintiles from quintile 1 to 5 (breast cancer). The number of enhancers is indicated in each case. **(H)** Proportion of mutations in gains, losses, and unchanged enhancers per cancer type. The number of mutations is displayed in each case. **(I)** Log transformed number of mutations normalized by enhancer width in thyroid cancer (two-sided Mann-Whitney *U*-test; $p = 5.59 \times 10^{-35}$ and $p = 3.59 \times 10^{-20}$ for the difference between gains and losses versus unchanged enhancers, respectively). n = 194, 557, and 97 gains, unchanged enhancers, and losses. **(J)** Log transformed number of mutations normalized by enhancer width in thyroid cancer across replication time quintiles (two-sided Mann-Whitney *U*-test**)**. n = 18, 24, 28, 63, and 73 gains in quintiles 1 to 5. n = 21, 53, 92, 187, and 287 unchanged enhancers in quintiles 1 to 5. n = 10, 9, 15, 32, and 35 losses in quintiles 1 to 5**. (K)** Plot as **(I)** for prostate cancer (two-sided Mann-Whitney *U*-test; $p = 8.20 \times 10^{-172}$ and $p = 6.64 \times 10^{-147}$ for the difference between gains and losses versus unchanged enhancers; n = 24672, 5996, and 6814 gains, unchanged enhancers, and losses). **(L)** Log transformed number of mutations normalized by enhancer width in prostate gains, losses, and unchanged enhancers across replication time quintiles (two-sided Mann-Whitney *U*-test). P-values for the comparison of gains and losses versus unchanged enhancers: p = 2.46 × 10-07 and p = 0.15 for quintile 1, $p = 6.4 \times 10^{-07}$ and $p = 2.8 \times 10^{-04}$ for quintile 2, $p = 2.03 \times 10^{-21}$ and $p = 1.58 \times 10^{-22}$ for quintile 3, $p = 7.79 \times 10^{-41}$ and $p = 4.09 \times 10^{-44}$ for quintile 4, $p = 2.27 \times 10^{-61}$ and $p = 1.02 \times 10^{-80}$ for quintile 5. n = 2657, 3884, 4784, 5814, and 7547 gains in quintiles 1 to 5. n = 120, 367, 857, 1517, and 3138 unchanged enhancers in quintiles 1 to 5. n = 370, 699, 1046, 1489, and 3212 losses in quintiles 1 to 5. **(M-N)** Median log-transformed number of mutations normalized by enhancer width in thyroid cancer **(M)** and AML **(N)** gains, losses, and unchanged enhancers across replication time quintiles. Error bars represent standard error. n = 194, 555, and 97 gains, unchanged enhancers and losses for thyroid cancer. n = 592, 876, and 303 for AML. The replication time shown for thyroid and AML was predicted from ATAC-seq data (predicted from thyroid and pre-leukemic HSCs, respectively). Significance notation: 'ns' $P > 0.05$; '***' $1 \times 10^{-4} < P \leq 1 \times 10^{-3}$; '****' $P \leq 1 \times 10^{-4}$. The total number of enhancers and mutations in each cancer type can be found in Supplementary Table 2.

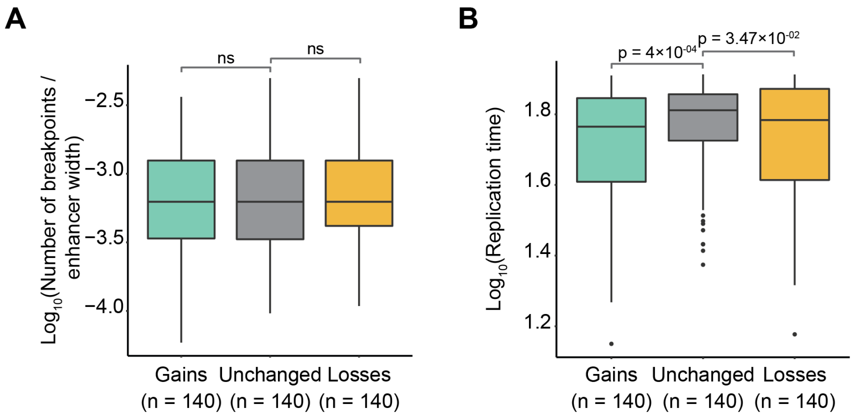

Supplementary Figure 19. Replication time in prostate enhancers matched to recombination breakpoints

(A) Number of recombination breakpoints is matched after normalizing by enhancer width for gained, unchanged, and lost enhancers. Number of enhancers is indicated (two-sided Mann-Whitney *U*-test, significance code ‘ns’ $p > 0.05$). **(B)** Log_{10} transformed mean replication time of the enhancers shown in **(A)** (one-sided Mann-Whitney *U*-test, unchanged enhancers vs. gains or losses, alternative = “greater,” p-value is indicated for every comparison).

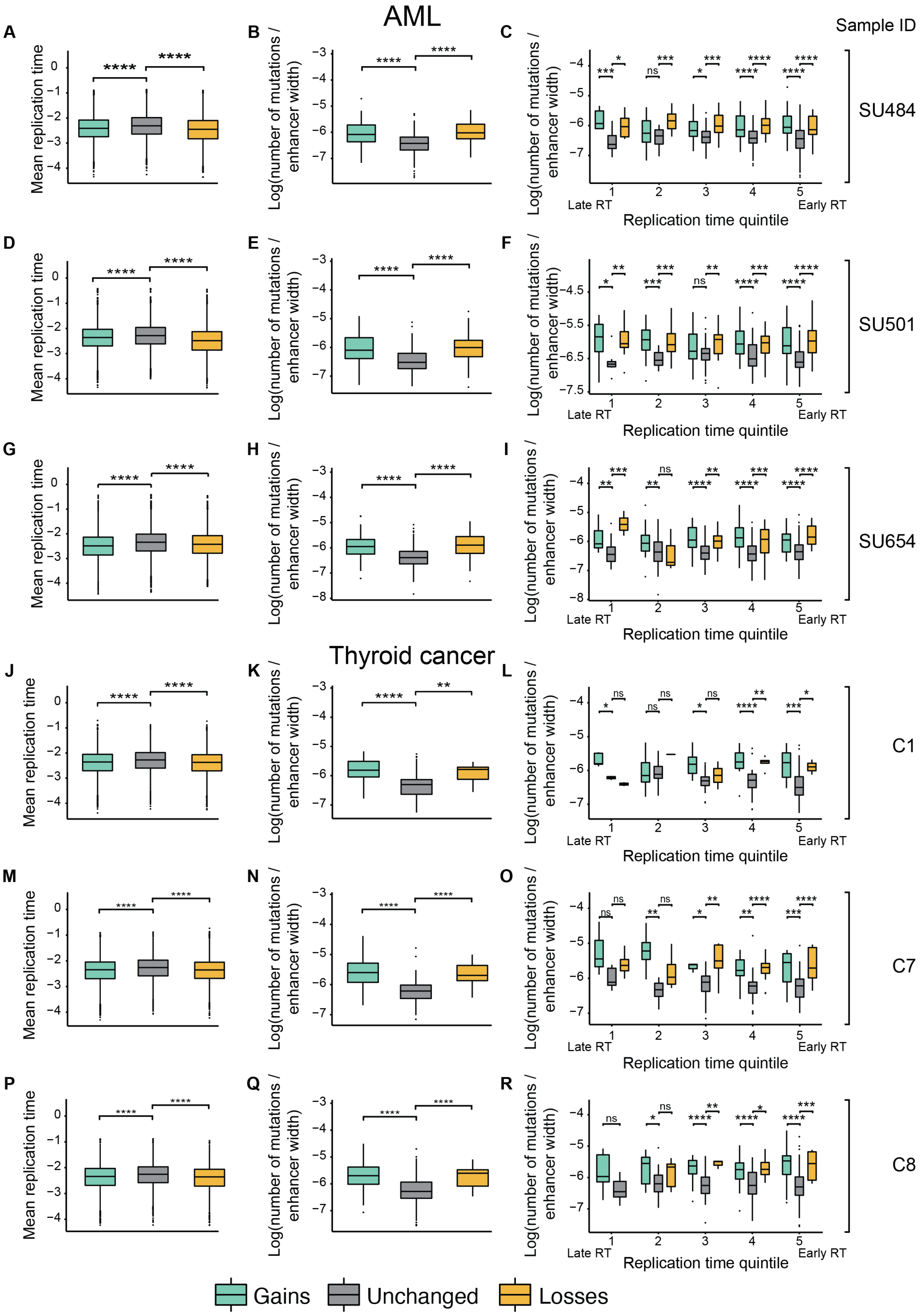

Supplementary Figure 20. Enhancer mutations in individual cancer samples
Mean replication time and number of mutations in AML **(A-I)** and thyroid cancer **(J-R)** gains, losses and unchanged enhancers (n = 3 AML and n = 3 thyroid cancer patient samples). The first column of plots shows the mean replication time of gains, losses, and unchanged enhancers in individual AML and thyroid cancer patient samples (sample ID indicated on the right of the figure) (Mann-Whitney *U*-test). Second column shows the log-transformed number of mutations normalized by enhancer width in AML and thyroid cancer enhancers (Mann-Whitney *U*-test). Column 3 indicated the log transformed number of mutations normalized by enhancer width across replication time quintiles (Mann-Whitney *U*-test). 'ns' denotes p > 0.05, '****' P ≤ 0.0001. The number of enhancers and mutations are indicated in Supplementary Table 2.

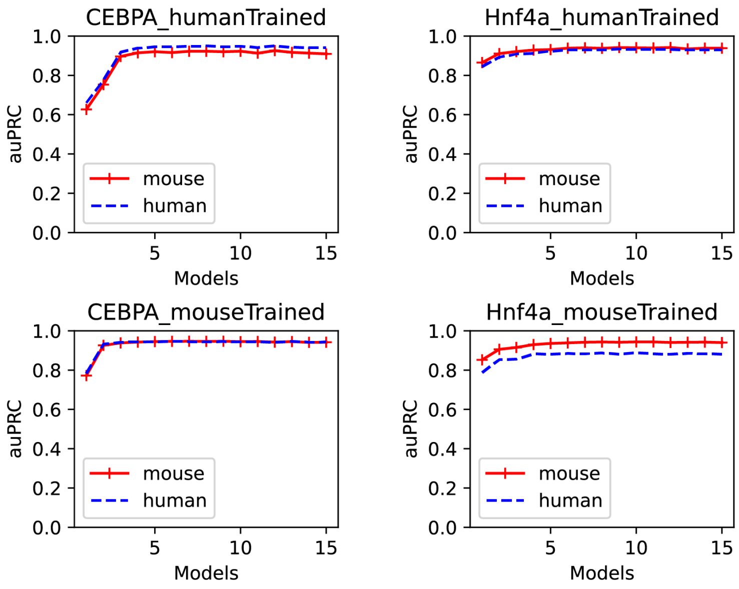

Supplementary Figure 21. Model performance on validation datasets

The figure shows area under the Precision Recall curve (auPRC) values for models predicting CEBPA or HNF4A binding sites on the validation sets. Results from the models trained on human and mouse data are shown on the top and bottom rows, respectively. Model numbers are on the X-axis.

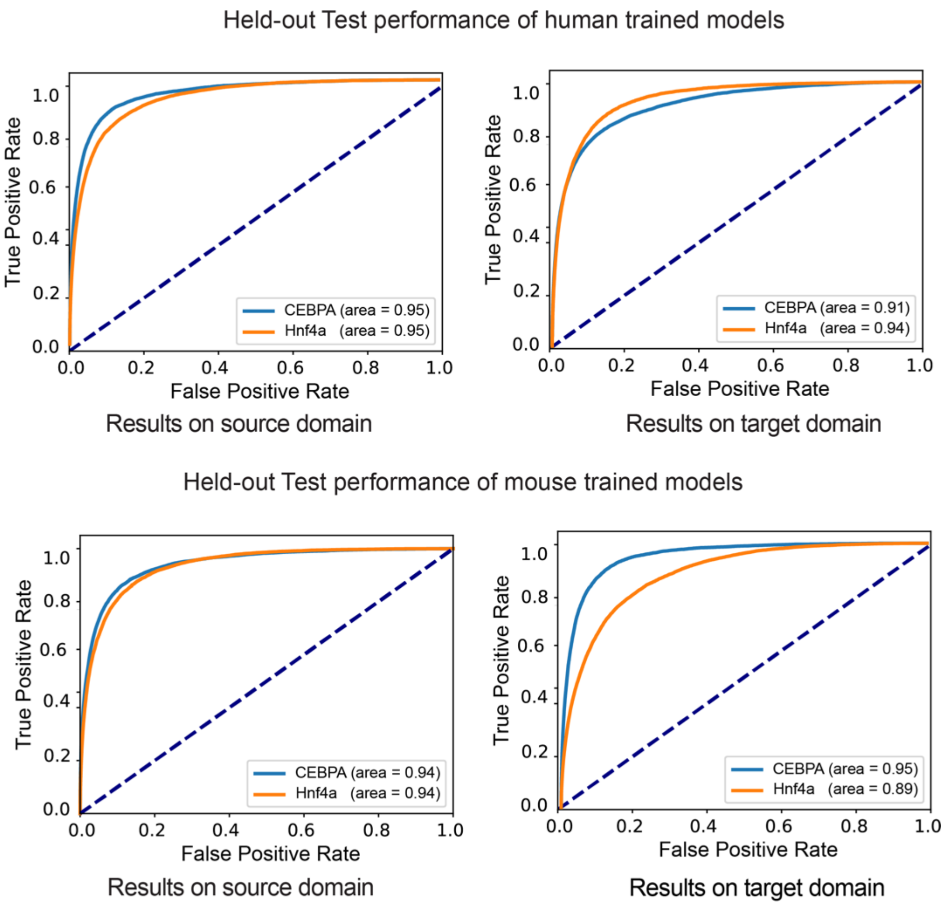

Supplementary Figure 22. Held-out Test performance of the trained models

Receiver Operating Characteristic (ROC) curves depict the performance of models trained on human (top) and mouse data (bottom). In the plots, ROC curves for CEBPA and Hnf4a are shown in blue and orange, respectively. The corresponding areas under the ROC curve values are indicated. Predictions made on the same species are labelled as “source domain”, while “target domain” denotes the performance on the data from the other species (mouse or human).

Supplementary Tables

Supplementary Table 1. Fisher test for the differential overlap of recent and conserved enhancers with accessible genomic regions

Fisher's tests were one-sided (alternative = "greater").

Species	Tissue	N conserved overlap accessible regions	N conserved do not overlap accessible regions	N recent overlap accessible regions	N recent do not overlap accessible regions	P-value	Odds ratio	Accession
**Human**	Liver	5119	8210	4451	5983	1	0.84	GSE170971
**Mouse**	Brain	3002	31782	3125	19185	1	0.58	ENCSR000COF
**Mouse**	Liver	2187	24851	4806	19990	1	0.37	ENCSR000CNI
**Mouse**	Muscle	671	32822	856	21761	1	0.52	ENCSR000CNX

Supplementary Table 2. Number of enhancers and mutations per cancer type

Cancer type	Sample	Enhancer type	Total number of enhancers	Number of mutations
**Prostate**		Gain	159821	38315
**Prostate**		Unchanged	40742	8176
**Prostate**		Loss	68984	8579
**Breast**		Gain	119844	NA
**Breast**		Unchanged	36413	NA
**Breast**		Loss	39114	NA
**Thyroid**	C1	Gain	14689	44
**Thyroid**	C1	Unchanged	19014	118
**Thyroid**	C1	Loss	15138	13
**Thyroid**	C7	Gain	11142	36
**Thyroid**	C7	Unchanged	16928	132
**Thyroid**	C7	Loss	18665	63
**Thyroid**	C8	Gain	22378	114
**Thyroid**	C8	Unchanged	18915	307
**Thyroid**	C8	Loss	7163	21
**Thyroid**	Union	Gain	41061	201
**Thyroid**	Union	Unchanged	28437	587
**Thyroid**	Union	Loss	34241	98
**AML**	SU484	Gain	25297	185
**AML**	SU484	Unchanged	18532	222
**AML**	SU484	Loss	28276	111
**AML**	SU501	Gain	34337	266
**AML**	SU501	Unchanged	24174	234
**AML**	SU501	Loss	31127	139
**AML**	SU654	Gain	27277	141
**AML**	SU654	Unchanged	40412	420
**AML**	SU654	Loss	16300	53
**AML**	Union	Gain	58875	627
**AML**	Union	Unchanged	45184	923
**AML**	Union	Loss	48249	318

Supplementary Table 3. Kruskal-Wallis' tests output for the difference in GC% across DNA replication time quintiles in Fig. 5A

	Chi-squared	p-value
**Enhancers**	2708.6	$< 2.2 \times 10^{-16}$
**Promoters**	688.25	1.22×10^{-147}
**Random regions**	902.98	3.76×10^{-194}

Supplementary Table 4. Number of regions per DNA replication time quintile in Fig. 5B

Region	DNA replication timing quintile	Number of regions
**Intergenic**	1	18542
**Intergenic**	2	24538
**Intergenic**	3	38575
**Intergenic**	4	55465
**Intergenic**	5	73896
**Exons**	1	7069
**Exons**	2	9325
**Exons**	3	16285
**Exons**	4	25966
**Exons**	5	37702
**Enhancers**	1	2212
**Enhancers**	2	2428
**Enhancers**	3	4771
**Enhancers**	4	7495
**Enhancers**	5	10586

Supplementary Table 5. Number of enhancers in Fig. 6

Group	Number of gains	Number of unchanged enhancers	Number of losses
**Prostate cancer**	159204	40658	68566
**Breast cancer**	119841	36413	39113
**AML**	592	876	303
**AML (quintile 1)**	35	45	26
**AML (quintile 2)**	73	72	39
**AML (quintile 3)**	122	168	67

**AML (quintile 4)**	176	238	98
**AML (quintile 5)**	186	353	73
**Prostate cancer (quintile 1)**	2657	120	370
**Prostate cancer (quintile 2)**	3884	367	699
**Prostate cancer (quintile 3)**	4784	857	1046
**Prostate cancer (quintile 4)**	5814	1517	1489
**Prostate cancer (quintile 5)**	7547	3138	3212

Supplementary Table 6. Mean human and mouse enhancers width

The mean width and frequency of human and mouse enhancers is shown. Values are separated by enhancer type, mark and tissue where enhancers are functional.

Species	Class	Tissue	Mark	N enhancers	Mean width (bp)
**Human**	Conserved	Liver	Active	13329	3632
**Human**	Recent	Liver	Active	10434	3072
**Mouse**	Conserved	Liver	Active	13255	2977
**Mouse**	Recent	Liver	Active	8283	2029
**Mouse**	Conserved	Liver	Poised	15892	1223
**Mouse**	Recent	Liver	Poised	16513	785
**Mouse**	Conserved	Brain	Active	19146	2585
**Mouse**	Recent	Brain	Active	8804	2034
**Mouse**	Conserved	Brain	Poised	18002	1229
**Mouse**	Recent	Brain	Poised	13506	877
**Mouse**	Conserved	Muscle	Active	18616	2815
**Mouse**	Recent	Muscle	Active	8947	2154
**Mouse**	Conserved	Muscle	Poised	17444	1284
**Mouse**	Recent	Muscle	Poised	13670	880
**Mouse**	Conserved	Testis	Active	8381	2899
**Mouse**	Recent	Testis	Active	5584	1698
**Mouse**	Conserved	Testis	Poised	14365	1201
**Mouse**	Recent	Testis	Poised	18626	768

References

Grant CE, Bailey TL, Noble WS. 2011. FIMO: scanning for occurrences of a given motif. *Bioinforma Oxf Engl* **27**: 1017–1018.

Madeira F, Pearce M, Tivey ARN, Basutkar P, Lee J, Edbali O, Madhusoodanan N, Kolesnikov A, Lopez R. 2022. Search and sequence analysis tools services from EMBL-EBI in 2022. *Nucleic Acids Res* **50**: W276–W279.

Schmidt D, Wilson MD, Ballester B, Schwalie PC, Brown GD, Marshall A, Kutter C, Watt S, Martinez-Jimenez CP, Mackay S, et al. 2010. Five-vertebrate ChIP-seq reveals the evolutionary dynamics of transcription factor binding. *Science* **328**: 1036–1040.

Weirauch MT, Yang A, Albu M, Cote AG, Montenegro-Montero A, Drewe P, Najafabadi HS, Lambert SA, Mann I, Cook K, et al. 2014. Determination and inference of eukaryotic transcription factor sequence specificity. *Cell* **158**: 1431–1443.

Zhou L, Feng T, Xu S, Gao F, Lam TT, Wang Q, Wu T, Huang H, Zhan L, Li L, et al. 2022. ggmsa: a visual exploration tool for multiple sequence alignment and associated data. *Brief Bioinform* **23**: bbac222.
