# Supplementary material for: Emergence of enhancers at late DNA replicating regions
Source: Nat Commun. 2024 Apr 24;15:3451. doi: 10.1038/s41467-024-47391-5 (PMC11043393; doi:10.1038/s41467-024-47391-5)
Supplement: Supplementary file 3 — Description of Additional Supplementary Files [file 41467_2024_47391_MOESM3_ESM.pdf]

Description of Additional Supplementary Data

File Name: **Supplementary Data 1**
Description: Gaussian Mixture model's output of the DNA replication time of TF binding sites in the cell line K562. ENCODE Accession numbers of the 71 TF binding datasets (ChIP-seq) are shown. mu1 and mu2 represent the mean replication time values of the two Gaussian components generated with each TF binding dataset. Likewise, sd1 and sd2 represent the standard deviation of the components, l1 and l2 represent the components' mixing proportions**,** and max1 and max2 represent the maximum value in every component.

File Name: **Supplementary Data 2**
Description: The number of human liver enhancers that show conserved activity in other species, those aligned to another species but without conserved activity, and those not alignable. Species symbols are defined in Supplementary Fig.1.
