# Supplementary material for: Emergence of enhancers at late DNA replicating regions
Source: Nat Commun. 2024 Apr 24;15:3451. doi: 10.1038/s41467-024-47391-5 (PMC11043393; doi:10.1038/s41467-024-47391-5)
Supplement: Supplementary file 6 — Reporting Summary [file 41467_2024_47391_MOESM6_ESM.pdf]

nature portfolio

Corresponding author(s): Wong E

Last updated by author(s): 25/2/24

Reporting Summary

Nature Portfolio wishes to improve the reproducibility of the work that we publish. This form provides structure for consistency and transparency in reporting. For further information on Nature Portfolio policies, see our Editorial Policies and the Editorial Policy Checklist.

Statistics

For all statistical analyses, confirm that the following items are present in the figure legend, table legend, main text, or Methods section.

n/a	Confirmed	
☐	☒	The exact sample size (*n*) for each experimental group/condition, given as a discrete number and unit of measurement
☐	☒	A statement on whether measurements were taken from distinct samples or whether the same sample was measured repeatedly
☐	☒	The statistical test(s) used AND whether they are one- or two-sided<br>*Only common tests should be described solely by name; describe more complex techniques in the Methods section.*
☐	☒	A description of all covariates tested
☐	☒	A description of any assumptions or corrections, such as tests of normality and adjustment for multiple comparisons
☐	☒	A full description of the statistical parameters including central tendency (e.g. means) or other basic estimates (e.g. regression coefficient) AND variation (e.g. standard deviation) or associated estimates of uncertainty (e.g. confidence intervals)
☐	☒	For null hypothesis testing, the test statistic (e.g. *F*, *t*, *r*) with confidence intervals, effect sizes, degrees of freedom and *P* value noted<br>*Give P values as exact values whenever suitable.*
☒	☐	For Bayesian analysis, information on the choice of priors and Markov chain Monte Carlo settings
☒	☐	For hierarchical and complex designs, identification of the appropriate level for tests and full reporting of outcomes
☐	☒	Estimates of effect sizes (e.g. Cohen's *d*, Pearson's *r*), indicating how they were calculated

Our web collection on statistics for biologists contains articles on many of the points above.

Software and code

Policy information about availability of computer code

Data collection	atll the data collection/data analysis software/tools/algorithms/packages used in the study are clearly mentioned in the manuscript.<br>Specific tools:<br>GATK v4.2.5.0<br>samtools v.1.10<br>R package mixtools version 1.2.0<br>R package “gkmSVM” version 0.83.0<br>HOMER v4.11<br>picard v2.26.10<br>RepeatMasker v4.0.6, v4.1.0<br>USCS liftOver tool<br>R v4.0.0
Data analysis	All methods, tools, version numbers are described in our methods and code to reproduce results are found on our GitHub page. Code used in analysis can be found on our GitHub page. https://github.com/ewonglab/enhancer_turnover

For manuscripts utilizing custom algorithms or software that are central to the research but not yet described in published literature, software must be made available to editors and reviewers. We strongly encourage code deposition in a community repository (e.g. GitHub). See the Nature Portfolio guidelines for submitting code & software for further information.

Data

Policy information about availability of data

All manuscripts must include a data availability statement. This statement should provide the following information, where applicable:

- Accession codes, unique identifiers, or web links for publicly available datasets
- A description of any restrictions on data availability
- For clinical datasets or third party data, please ensure that the statement adheres to our policy

All data accessions are provided and referenced in the manuscript.
Processed datasets used in this study are deposited to Zenodo doi:10.5281/zenodo.10494781.
Human and mouse-specific ChIP-seq original data are available under accessions E-MTAB-2633 [https://www.ebi.ac.uk/biostudies/arrayexpress/studies/E-MTAB-2633] and E-MTAB-7127 [https://www.ebi.ac.uk/biostudies/arrayexpress/studies/E-MTAB-7127]. H9 DNA replication time is available under accession GSE137764 [https://www.ncbi.nlm.nih.gov/geo/query/acc.cgi?acc=GSE137764]. Mouse PGC and SSC DNA replication times are available under accession GSE109804 [https://www.ncbi.nlm.nih.gov/geo/query/acc.cgi?acc=GSE109804]. Mouse somatic DNA replication times are available under accession GSE18019 [https://www.ncbi.nlm.nih.gov/geo/query/acc.cgi?acc=GSE18019]. ChIP-seq data from the prostate cancer cell line and prostate epithelial cells (PrEC) used in the study are available under accessions GSE73783 [https://www.ncbi.nlm.nih.gov/geo/query/acc.cgi?acc=GSE73783], GSE57498 [https://www.ncbi.nlm.nih.gov/geo/query/acc.cgi?acc=GSE57498]. ChIP-seq data of histone marks in the breast cancer cell line, MCF-7, and healthy epithelial breast cells are available under accessions GSE96352 [https://www.ncbi.nlm.nih.gov/geo/query/acc.cgi?acc=GSE96352], GSE86714 [https://www.ncbi.nlm.nih.gov/geo/query/acc.cgi?acc=GSE86714], and GSE139697 [https://www.ncbi.nlm.nih.gov/geo/query/acc.cgi?acc=GSE139697], GSE139733 [https://www.ncbi.nlm.nih.gov/geo/query/acc.cgi?acc=GSE139733], respectively. Matched ATAC-seq from cancer and healthy thyroid samples from three individuals are available at GSE162515 [https://www.ncbi.nlm.nih.gov/geo/query/acc.cgi?acc=GSE162515] (C1, C7, C8). ATAC-seq files for the matched pre-leukemic and blast cells from three individuals are available at GSE74912 [https://www.ncbi.nlm.nih.gov/geo/query/acc.cgi?acc= GSE74912] (SU484, SU501, SU654).

Databases used include:
funMotifs (v1.0),JASPAR 2020 ,RepBase (v27.04),

Research involving human participants, their data, or biological material

Policy information about studies with human participants or human data. See also policy information about sex, gender (identity/presentation), and sexual orientation and race, ethnicity and racism.

Reporting on sex and gender	NA
Reporting on race, ethnicity, or other socially relevant groupings	NA
Population characteristics	NA
Recruitment	NA
Ethics oversight	NA

Note that full information on the approval of the study protocol must also be provided in the manuscript.

Field-specific reporting

Please select the one below that is the best fit for your research. If you are not sure, read the appropriate sections before making your selection.

☒ Life sciences ☐ Behavioural & social sciences ☐ Ecological, evolutionary & environmental sciences

For a reference copy of the document with all sections, see nature.com/documents/nr-reporting-summary-flat.pdf

Life sciences study design

All studies must disclose on these points even when the disclosure is negative.

Sample size	All applicable and available data was used. We tried to maximize the number of datasets we used while ensuring each dataset was robust. Data from multiple species, tissues, and cancer types were used. Where cancer samples from matched healthy and cancer primary samples were used, analyses were performed using samples taken from three patients.
Data exclusions	All regions that could be aligned were included in the study
Replication	Multi-species ChIP-seq datasets used were replicated (at least 3 biological replicates) and multiple filtering steps were taken to ensure robustness as described in the manuscript. In our cancer analyses, we used data from multiple individuals, matched in tumour and healthy tissue samples to ensure accuracy in the calling of somatic variants from 3 individuals.

Randomization	NA as analyses was performed using publicly available data.
Blinding	NA as analyses was performed using publicly available data.

Reporting for specific materials, systems and methods

We require information from authors about some types of materials, experimental systems and methods used in many studies. Here, indicate whether each material, system or method listed is relevant to your study. If you are not sure if a list item applies to your research, read the appropriate section before selecting a response.

Materials & experimental systems

n/a	Involved in the study
☒	☐ Antibodies
☒	☐ Eukaryotic cell lines
☒	☐ Palaeontology and archaeology
☒	☐ Animals and other organisms
☒	☐ Clinical data
☒	☐ Dual use research of concern
☒	☐ Plants

Methods

n/a	Involved in the study
☒	☐ ChIP-seq
☒	☐ Flow cytometry
☒	☐ MRI-based neuroimaging

Plants

Seed stocks	NA
Novel plant genotypes	NA
Authentication	NA
